# Role of the epithelial barrier in intestinal fibrosis associated with inflammatory bowel disease: relevance of the epithelial-to mesenchymal transition

**DOI:** 10.3389/fcell.2023.1258843

**Published:** 2023-09-26

**Authors:** Dulce C. Macias-Ceja, M. Teresa Mendoza-Ballesteros, María Ortega-Albiach, M. Dolores Barrachina, Dolores Ortiz-Masià

**Affiliations:** ^1^ Departamento de Farmacología and CIBEREHD, Facultad de Medicina, Universidad de Valencia, Valencia, Spain; ^2^ Instituto de Educación Superior Isabel de Villena, Conselleria de Educación, Cultura y Deporte, Valencia, Spain; ^3^ INCLIVA Biomedical Research Institute, Valencia, Spain; ^4^ Departamento de Medicina, Facultad de Medicina, Universidad de Valencia, Valencia, Spain

**Keywords:** fibrosis, epithelial cells, intestinal fibrosis, inflammatory bowel disease, crohn, colitis, epithelial mesenchymal transition

## Abstract

In inflammatory bowel disease (IBD), chronic inflammation in the gastrointestinal tract can lead to tissue damage and remodelling, which can ultimately result in fibrosis. Prolonged injury and inflammation can trigger the activation of fibroblasts and extracellular matrix (ECM) components. As fibrosis progresses, the tissue becomes increasingly stiff and less functional, which can lead to complications such as intestinal strictures, obstructive symptoms, and eventually, organ dysfunction. Epithelial cells play a key role in fibrosis, as they secrete cytokines and growth factors that promote fibroblast activation and ECM deposition. Additionally, epithelial cells can undergo a process called epithelial-mesenchymal transition, in which they acquire a more mesenchymal-like phenotype and contribute directly to fibroblast activation and ECM deposition. Overall, the interactions between epithelial cells, immune cells, and fibroblasts play a critical role in the development and progression of fibrosis in IBD. Understanding these complex interactions may provide new targets for therapeutic interventions to prevent or treat fibrosis in IBD. In this review, we have collected and discussed the recent literature highlighting the contribution of epithelial cells to the pathogenesis of the fibrotic complications of IBD, including evidence of EMT, the epigenetic control of the EMT, the potential influence of the intestinal microbiome in EMT, and the possible therapeutic strategies to target EMT. Finally we discuss the pro-fibrotic interactions epithelial-immune cells and epithelial-fibroblasts cells.

## 1 Introduction

Inflammatory bowel disease (IBD) is a complex condition influenced by a combination of genetic, environmental, and immunological factors. Environmental factors, such as changes in diet, increased hygiene practices, and alterations in the gut microbiota, are believed to play a role in the development of IBD. The two main types of IBD are Crohn’s disease (CD) and ulcerative colitis (UC). CD is characterized by inflammation that can occur anywhere in the gastrointestinal (GI) tract, although it affects most commonly the small intestine and the right colon. The inflammation in CD involves multiple layers of the bowel wall. On the other hand, UC is limited to the colon and the rectum. Both, CD and UC, are chronic conditions characterised by periods of active disease and periods of remission, and the eventual development of intestinal fibrosis. This inevitable progression towards fibrosis suggests that fibrosis becomes inflammation-independent and auto-propagative (Santacroce et al., 2022; Park et al., 2023). The course and extent of fibrosis show significant variability between individual patients, indicating a genetic component (Jarmakiewicz-Czaja et al., 2023; Macias-Ceja et al., 2023). In CD, approximately 50% of patients develop fibrotic strictures or penetrating lesions (Cosnes et al., 2011) and it is estimated that up to 70% of patients will eventually require surgery at some point during their disease course (Yoo et al., 2020). Despite undergoing surgical interventions, it is not uncommon for patients to experience post-operative recurrence of fibrosis, particularly at the site of an ileocolonic anastomosis. This recurrence can lead to the development of re-stricturing disease, potentially necessitating additional surgeries (Gklavas et al., 2017). The incidence of intestinal strictures in CD has not significantly changed, as current anti-inflammatory therapies neither prevent nor reverse the established fibrosis/strictures, indicating that control of inflammation does not essentially affect the fibrotic course.

Intestinal fibrosis involves the accumulation of extracellular matrix (ECM) components in the intestinal wall, and this process is driven by activated cells of mesenchymal source, including fibroblasts, myofibroblasts, and smooth muscle cells. The deposition of ECM differs between UC and CD: in UC, fibrosis is primarily restricted to the superficial layers of the intestine (mucosal and submucosal layers) (Gordon et al., 2014), while fibrosis in CD occurs mainly in the ileocecal valve and can affect the entire thickness of the bowel wall due to the transmural nature of the inflammation (Yoo et al., 2020). Currently, one of the main goals of IBD treatment is to induce wound healing. Mucosal healing is a biological process activated by inflammation that is capable, depending on the equilibrium between production/degradation of the ECM component, of either restoring the integrity of the damaged epithelial barrier with reconstitution of normal intestinal function or triggering fibrosis (D’Haens et al., 2022; Otte et al., 2023).

Various factors contribute to the development and progression of fibrosis in IBD. Soluble molecules, including growth factors and cytokines, play a significant role, with transforming growth factor-beta 1 (TGFβ1) being considered a key player. These molecules are released by activated immune and nonimmune cells and contribute to the activation of fibroblasts and myofibroblasts, leading to excessive ECM production and fibrotic remodelling. In addition to soluble molecules, other mechanisms involved in intestinal fibrosis include G protein-coupled receptors, the gut microbiota and epithelial-to-mesenchymal transition (EMT) or endothelial-to-mesenchymal transition (EndoEMT), which are processes where epithelial or endothelial cells acquire a mesenchymal phenotype and contribute to fibrosis (D’Alessio et al., 2022).

Since fibrosis can progress once established, regardless of whether inflammation is suppressed or not, antifibrotic drugs are now targeting mechanisms that are independent of inflammation (Solitano et al., 2023). Science has focused on various aspects, including the inflammation-independent mechanisms behind the gut fibrotic process (Zhao et al., 2020), or environmental (Amamou et al., 2022) and genetic risk factors (Macias-Ceja et al., 2023). This has led to a deeper exploration of aspects such as aberrant wound healing, dysregulated extracellular matrix production, and activation of specific cell types (such as fibroblasts) that promote fibrogenesis (Yoo et al., 2020). The traditional opinion that intestinal fibrosis is an irreversible process is changing in light of an improved understanding of the cellular and molecular mechanisms that underline the pathogenesis of fibrosis.

Epithelial cells are involved in the fibrotic process at both the cellular and molecular level. They can secrete cytokines and growth factors, such as TGFβ and platelet-derived growth factor (PDGF), that promote the activation of fibroblasts or extend the pool of mesenchymal cells through the EMT process. The interactions between epithelial cells, immune cells, and fibroblasts form a complex network that promotes the development and progression of fibrosis in IBD. In this review, we have collected and discussed the recent literature highlighting the contribution of epithelial cells to the pathogenesis of the fibrotic complications of IBD, including evidence of EMT, the epigenetic control of EMT, the potential influence of the intestinal microbiome in EMT, and the possible therapeutic strategies to target EMT. Finally, we discuss the pro-fibrotic interactions between epithelial cells, immune cells, and fibroblasts.

## 2 A brief outline of the epithelial barrier role in the pathophysiology of IBD

Under normal and homeostasis conditions, the intestinal epithelial barrier comprises a thick mucosal layer that is associated with specialized intestinal epithelial cells (IECs) linked together by tight junctions (TJs) and resident microbiota, collectively forming a healthy layer. Intestinal stem cells, located in the base of the crypt, divide and differentiate to give rise to five different types of IECs (enterocytes, Paneth cells, goblet cells (GCs), enteroendocrine cells and microfold cells) maintaining the integrity of the intestinal epithelium.

**FIGURE 1:**
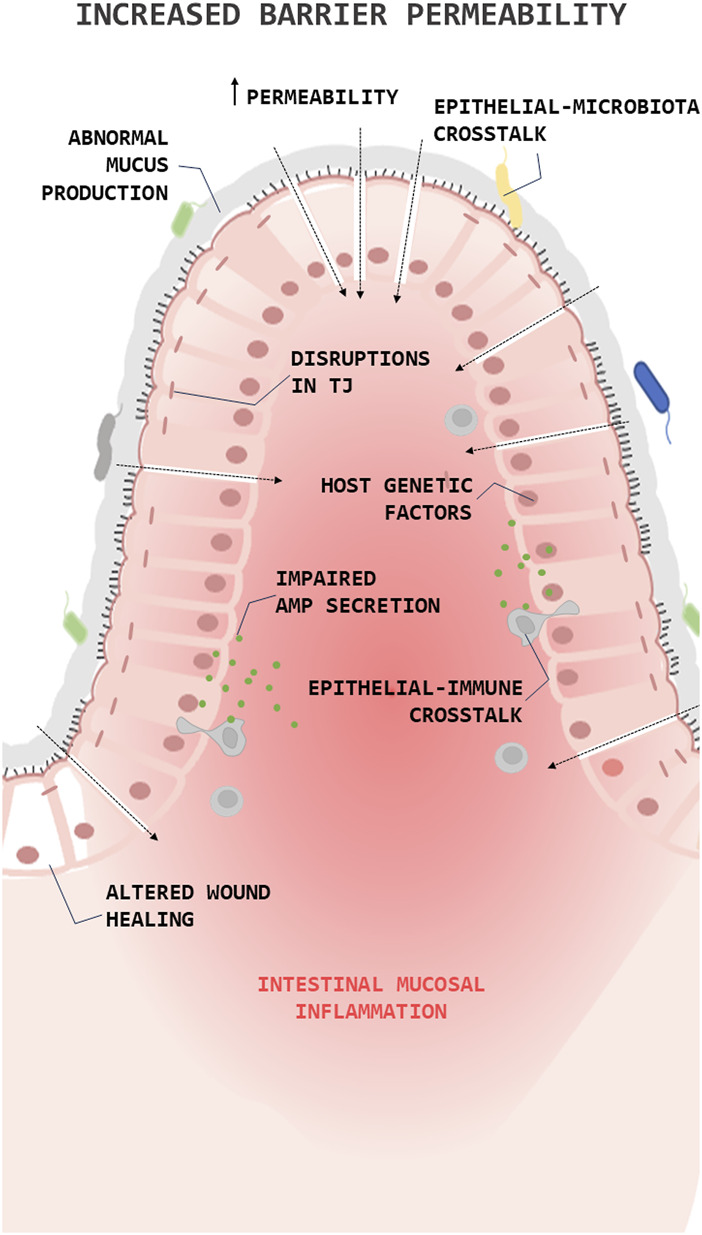
Role of the epithelial barrier in the pathophysiology of Inflammatory Bowel Diseases (IBDs). Simplified illustration of the role of epithelial barrier in the pathophysiology of IBD. The illustration shows the main processes in which epithelial cells are involved in the pathogenesis of IBD. Antimicrobial peptide (AMP); Tight junction (TJ).

It is known that the loss of barrier integrity and the increase in overall barrier permeability are fundamental processes in the IBD pathophysiology (barrier loss activates immunoregulatory processes) (Kotla and Rochev, 2023). The barrier loss can be triggered by various factors, including disruptions in the tight junction, abnormal mucus production, impaired antimicrobial peptide (AMPs) secretion, altered wound healing, or environmental and genetic factors (Figure 1). In IBD, the high levels of proinflammatory (T helper type 1 (Th1)) cytokines (Tumor necrosis factor alpha (TNFα), Interleukin (IL)1β, and IL6) change the composition of the TJs decreasing transepithelial electrical resistance and amplify mucosal inflammation (Lee, 2015). Along this line, clinical studies have shown a decrease in the expression and redistribution of the junctional complexes in both UC (Blair et al., 2006) and CD (Zeissig et al., 2007) patients.

Over the years, a series of studies showed that mucin expression/secretion by GCs (the primary secretory cells of the GI trac) is mediated by cytokines (both Th1 and Th2 cytokines), inflammasome related proteins (autophagy dysregulation), gut microbiota and the diet (Melhem et al., 2021). For instance, Western diets (high fat/high sugar) lead to endoplasmic reticulum stress and oxidative stress in GCs reducing the production/secretion of mucins (Gulhane et al., 2016) and alters microbial communities, improving the colonization of *E. coli* (Martinez-Medina et al., 2014) or favouring an overgrowth of pro-inflammatory bacteria, such as *Proteobacteria* (Agus et al., 2016). This, together with the fact that the products derived from bacteria can regulate the production and secretion of mucin, thus promoting the loss of the integrity of the epithelial barrier (Figure 1). Lastly, epithelial repair is known to be altered in IBD and this is reflected in the creation of aberrant intestinal anastomosis after a bowel resection, giving rise to the recurrence of the disease in the same place (Kelm and Anger, 2022).

Mucosal healing is a complex process that encompasses the migration/proliferation of IECs as well as regulation by gut microbial peptides, and growth factors (Alam and Neish, 2018), that can be altered by genetic and epigenetic factors. Genome-wide association studies (GWAS) have indicated risk alleles in IBD patients in genes involved in intestinal cell restitution (ERRFI1, PTGER4 or HNF4), in cell polarity (PARD3) or in intercellular junctions (MYO9B, MAGI2, GNAI2, LAMB1 or CDH1) (McCole, 2014). At a epigenetic level, long non coding RNA (ncRNA) CCAT1 and FBXL19-AS1 (Ma et al., 2019; Zhao et al., 2022), circular CDKN2B-AS1 and SMAD4 (Rankin et al., 2019; Zhao et al., 2023) and microRNAs (miR) miR-21, miR23a, miR-182-5p (Shi et al., 2013; Yang et al., 2013; Felwick et al., 2020; Xu et al., 2022) overexpression can increase the degradation of the epithelial barrier while miR-195-5p reduces the permeability (Scalavino et al., 2022).

Currently, the goal of IBD therapy (gold standard) in long remission is the epithelial repair and mucosal healing (Colombel et al., 2020; D’Arcangelo and Aloi, 2020). However, there are no approved therapies targeting the epithelium. There are indeed various approaches being explored in the field of epithelial research that have the potential to lead to new therapies, such as the use of epithelial stem cells, growth factors or cytokines, and modifications of the intestinal microbiota (Liu et al., 2021). It is worth noting that these approaches are still under active research and development. However, they represent exciting paths for potential therapeutic interventions in IBD.

## 3 EMT role in intestinal fibrosis

EMT, first described in 1995 (Hay, 1995), is a reversible process in which the characteristics of epithelial cells are modified until reaching the characteristics of mesenchymal cells, passing through intermediate characteristics between both cell types. In the literature, there are three types of EMT described: the ones associated with embryogenesis/development (type-1 EMT); the ones involved in wound healing (type-2 EMT); and the ones associated with cancer progression (type-3 EMT) (Marconi et al., 2021). Several studies have reported that damaged epithelial cells may act as crucial sources of fibroblasts and contribute to organ fibrosis through type-2 EMT in different fibrotic tissues (Tennakoon et al., 2015; Rout-Pitt et al., 2018; Ortiz-Masiá et al., 2020a; Macias-Ceja et al., 2022; Hadpech and Thongboonkerd, 2023) where specialized epithelial cells give rise to myofibroblasts with profibrotic and pro-inflammatory activity, which expresses α smooth muscle actin (α-SMA) and VIMENTIN but does not express epithelial markers, such as E-CADHERIN (CDH1), ZONULAE OCCLUDENTES (ZOS) or claudins. Various transcriptional factors regulate the process, such as SNAIL Family Transcriptional Repressor (SNAIL1/2), ZINC-FINGER E-BOX-BINDING (ZEB1/2), SLUG or TWIST transcription factors (TWIST1/2) (Xu et al., 2019).

Numerous studies support the role of EMT in the pathogenesis of intestinal fibrosis. In this section we will review the contribution of EMT to the pathogenesis of the fibrotic complications of IBD. Specifically, we review the evidence of EMT in patients, the molecular mechanisms involved, and the role of epigenetic and genetic. Finally, we discuss the role EMT as a therapeutic target in IBD.

### 3.1 Evidence of EMT in IBD patients and *in vivo* models

In IBD, EMT was observed for the first time in 2008 in the intestinal fistulae of CD patients (Bataille et al., 2008). From 2008 to 2023, several studies have revealed the presence of EMT markers in CD and UC patients (Table 1). In CD, the presence of EMT markers has been demonstrated in all disease phenotypes [Montreal classification (Satsangi et al., 2006)]: in transitional cells from entero-cutaneous surrounding fistulae, in fibrotic areas from fistulae, in stenotic tissues and in inflamed mucosa. In relation with type-3 EMT, SLUG expression has been related with tumor progression in CD (Scharl et al., 2014). Regarding UC, the literature about type-2 EMT in intestinal fibrosis is limited. The intestinal samples analysed in most studies do not specify the presence of fibrosis or are performed on inflamed tissue (Table 1). Penetrating or stricturing complications are more common in CD (Thia et al., 2010) than in UC (Yamagata et al., 2011). However, the core problem in UC is the risk of dysplasia/cancer (CD, 2.4%; UC, 10.0%) (Fumery et al., 2015). This could be an explanation about why studies in patients with UC are more directed towards type-3 EMT (Saito et al., 2011; Wang et al., 2012; Tahara et al., 2014; Zhao et al., 2015).

**TABLE 1:** Reports of EMT in inflammatory bowel diseases (IBDs). Alpha Smooth Muscle Actin (α-SMA); E-cadherin (CDH1); Crohn’s disease (CD); N-cadherin (CDH2); Epithelial growth factor (EGF); Fibroblast activation protein (FAP); Fibroblast growth factors (FGF); Fibronectin (FN); Matrix metalloproteinase (MMP); Transforming growth factor β (TGFβ); Tumour necrosis factor (TNF); Tumour necrosis factor receptor (TNFR); Zinc-finger E-box-binding (ZEB).

Intestinal fibrotic samples	EMT histological localization/EMT markers	References
CD N = 15 resections	**Entero-cutaneous fistula specimens** (+) CYTOKERATIN 8/20, β6-INTEGRIN, nuclear Β-CATENIN, TGFβ1/2 (−) VIMENTIN, CDH1	Bataille et al. (2008)
IBD N = ¿? resections	**Intestinal crypts** ↑ α-SMA/↓ CDH1 cells	Flier et al. (2010)
CD N = 7 resections	**Entero-cutaneous fistula specimens** (+) SNAIL, FGF1/2/4/7 (−) EGF/TWIST	Scharl et al. (2011)
	**Fibrotic lesions** (+) SLUG, TNF/TNFR1	
IBD N = 22 biopsies	**Inflamed mucosa** (+) N-CADHERIN, ↑VIMENTIN/↓CDH1 cells	Chen et al. (2013)
CD N = 18 biopsies/resections	**Fibrotic lesions** (+) nuclear Β-CATENIN, SLUG, FAP, TGFβ1	Scharl et al. (2015)
IBD N = 20 resections	**Inflamed mucosa** (+) SLUG/SNAIL	Zidar et al. (2016)
CD N = 26 resections	**Fibrotic lesions** ↑VIMENTIN ↓CDH1	Xu X et al. (2017)
IBD pediatric N = 44 biopsies	**Inflamed mucosa** ↑ SNAIL/↓CDH1	Pierdomenico et al. (2018)
IBD N = 10 biopsies	**Inflamed mucosa** ↑ SNAIL, ZEB2, VIMENTIN, MMP9, ↓CDH1	Boros et al. (2018)
CD N = 31biopsies	**Inflamed mucosa** ↑FSP1, VIMENTIN, nuclear Β-CATENIN, ↓CDH1	He et al. (2018)
CD N = 57 resections	**Fibrotic lesions/Entero-cutaneous fistula specimens/Intestinal crypts** ↑VIMENTIN/↓ CDH1cells ↑ SNAIL/SLUG, CDH2, DESMIN, ZEB1	Ortiz-Masià et al. (2020b)
IBD N = 16 resections	**Inflamed mucosa** ↑ SNAIL/SLUG	Ortiz-Masiá et al. (2020a)
IBD N = 32 resections	**Fibrotic lesions** ↑FSP1 and α-SMA/↓ CDH1cells (+) nuclear Β-CATENIN	Wenxiu et al. (2021)
CD N = 30 biopsies	**Fibrotic lesions** (+) CDH2,VIMENTIN, TIMP1, FN	Wang et al. (2022)
IBD N = 133 biopsies	**Inflamed mucosa** ↑ SNAIL, CDH2 ↓CDH1	Ghorbaninejad et al. (2022)
IBD N = 5 biopsies	**Inflamed mucosa** ↑VIMENTIN/↓ CDH1 cells, nuclear Β-CATENIN	Pompili et al. (2023)

Animal models have played a crucial role in advancing our understanding of intestinal fibrosis. Murine models of intestinal fibrosis include chemical induction (trinitrobenzene sulfonic acid (TNBS) or dextran sodium sulfate (DSS)), genetic manipulation (IL-10KO), ionizing radiation, or surgical techniques (hetero transplantation of small bowel) (Li et al., 2021). A very interesting animal model that has helped to gain insight into the EMT process in intestinal fibrosis are the VillinCre; R26Rosa-lox-STOP-lox-LacZ double transgenic mice, which have made it possible to track mesenchymal cells derived from epithelial cells (Flier et al., 2010). The presence of EMT markers in fibrotic mouse models has been widely demonstrated by Lovisa’s review (Lovisa et al., 2019). Two interesting findings from animal models are that one-third of the fibroblasts are derived from epithelial cells in the TNBS model (Flier et al., 2010), or that the cells that enter in EMT do not move and remain in their original anatomical location in the DSS model, favouring fibroblasts transdifferentiation through the release of profibrotic mediators (Zeng et al., 2022).

### 3.2 EMT in intestinal fibrosis: molecular mechanism

**FIGURE 2:**
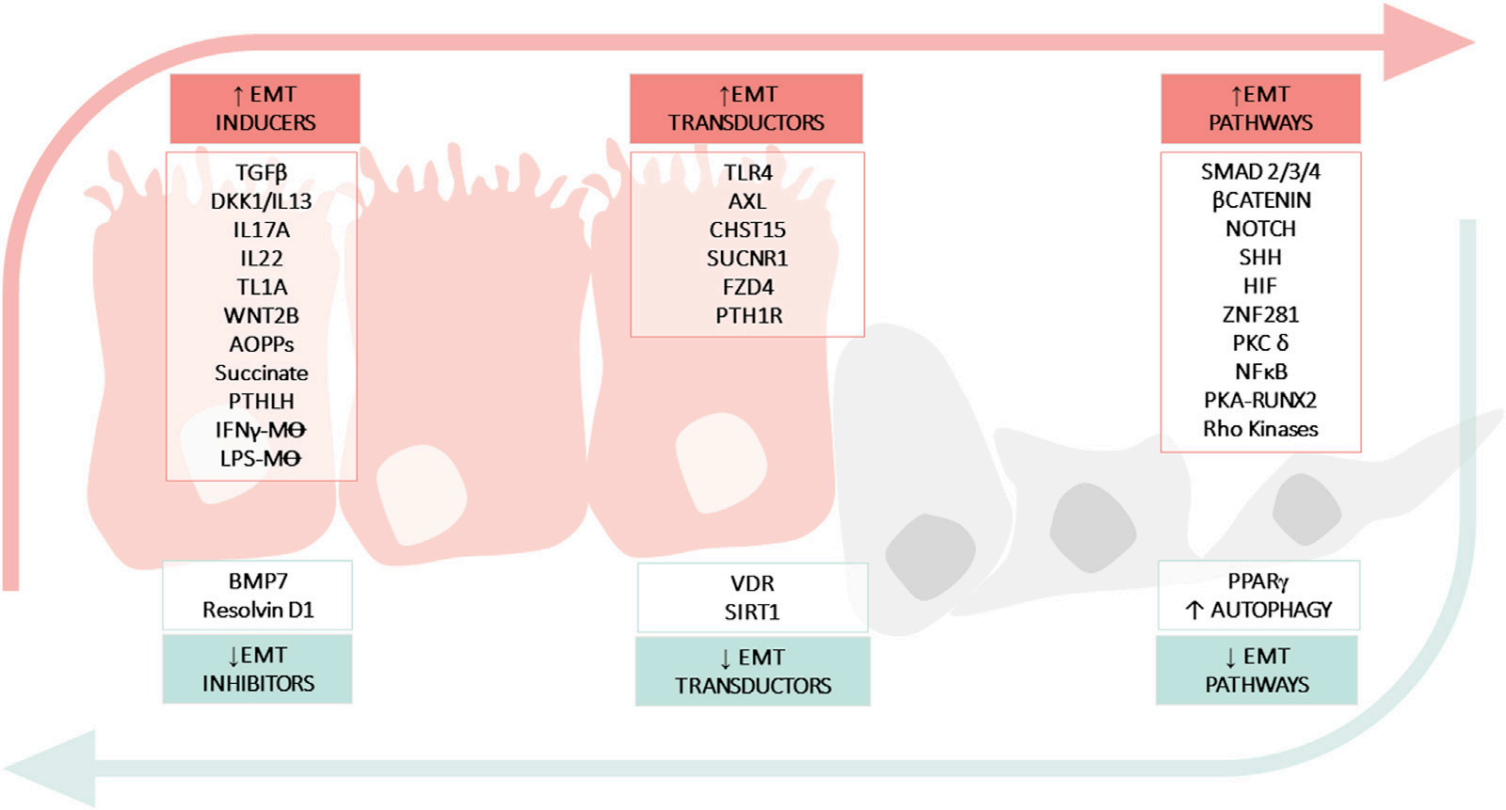
Molecular mechanisms implicated in epithelial mesenchymal transition (EMT) in IBD. The figure shows: soluble molecules, cells or hormones related with the induction (INDUCERS) or inhibition (INHIBITORS) of the EMT process; receptors or enzymes implicated in the EMT process (TRANSDUCTORS) and pathways related with EMT (PATHWAYS). Advanced oxidation protein products (AOPPs); Bone morphogenic protein-7 (BMP7); Carbohydrate sulfotransferase 15 (CHST15); Crohn’s Disease (CD); Dickkopf-homolog-1 (DKK1); Hypoxia inducible factor (HIF); Inflammatory Bowel disease (IBD); Interferon (IFN); Interleukin (IL); Lipopolysaccharide (LPS); Macrophage (M⊝); Nuclear Factor kB (NFκB); Toll-like receptor 4 (TLR4); Parathyroid hormone-like hormone (PTHLH); Parathyroid hormone receptor 1 (PTH1R); Protein kinase (PK); Peroxisome proliferator-activated receptor (PPARγ); Runt-related transcription factor 2 (Runx2); Silent information regulator 1 (SIRT1); Sonic Hedgehog (SHH); Transforming growth factor (TGF); Tumour necrosis factor-like ligand 1A (TL1A); Ulcerative Colitis (UC); Vitamin D receptor (VDR); Zinc-finger E-box-binding (ZEB).

Type-2 EMT is particularly observed in CD. Although the factors that drive type-2 EMT in IBD are not yet fully understood, various signalling pathways (TGFβ/SMAD, WNT, NOTCH, hypoxia-inducible factor-1α (HIF1α) and Hedgehog pathways) and molecules (growth factors, cytokines, proteases, oxidative stress, and hormones) have been implicated. In this section, we review the main pathways and molecules involved in the EMT-modulation of intestinal fibrosis associated with IBD (Table 2) (Figure 2).

**TABLE 2:** Molecular mechanism implicated in upregulation of type-2 EMT in IBD samples, and *in vivo* and *in vitro* IBD related models. The symbol “/” indicates treatment. Advanced oxidation protein products (AOPPs); Carbohydrate sulfotransferase 15 (CHST15); Crohn’s disease (CD); Bone morphogenic protein-7 (BMP7); Dextran sodium sulfate (DSS); Dickkopf-homolog-1 (DKK1); Intestinal epithelial cell (IEC); Interferon (IFN); lipopolysaccharide (LPS); Parathyroid hormone-like hormone (PTHLH); parathyroid hormone receptor 1 (PTH1R); protein kinase A (PKA); Runt-related transcription factor 2 (Runx2); Sonic Hedgehog (SHH); Trinitrobenzene sulfonic acid (TNBS); Tumour necrosis factor-like ligand 1A (TL1A); Transititonal cells lining the fistula tract (TC); Transforming growth factor (TGF); Interleukin (IL); Tumour necrosis factor (TNF); Toll-like receptor 4 (TLR4); Ulcerative colitis (UC); Zinc-finger E-box-binding (ZEB).

Protein	Type of study		EMT	Molecular mechanism in EMT	References
TGFβ	*In vitro*	HT29 cells/TGFβ	↑	IL13, SNAIL1	Scharl et al. (2013)
IL13	*In vitro*	HT29 cells/IL13	↑	SLUG	Scharl et al. (2013)
DKK1	*In vitro* Human	HT29 cells/TGFβ1 CD: ↑DKK1 in TC	↑	IL13	Frei et al. (2013)
TL1A	*In vitro In vivo* Human	HT29 cells/TL1A/BMP7 DSS TL1A overexpression IBD: ↑TL1A	↑	TGFβ/Smad3	Wenxiu et al. (2021)
IL22	*In vitro I n In vivo*	Caco-2, HT29 and T84 cells/IL22, TGFβ1, IFNγ, TNFα Toxoplasma model IL22^−/−^	↑	ERK	Delbue et al. (2021)
IL17A	*In vitro In vivo*	IEC6/IL17A Mouse intestine	↑	SNAIL	Zhang et al. (2018)
IFNγ	*In vitro* Human	HT29 cells cocultured with IFNγ -U937 macrophages CD: ↑IFNγ and IFNγ receptor	↑	WNT/FZD4	Macias-Ceja et al. (2022)
SHH	*In vitro* Human	Caco2 cells coculture with LPS-RAW264.7cells/HPI-1 or GANT-61 (HH inhibitors) IBD: ↑SHH activity	↑	SHH	Ghorbaninejad et al. (2022)
WNT2b/FZD4	*In vitro Human*	HT29 cells/WNT2b CD Biopsies/WNT2b CD: ↑WNT2B/FZD4	↑	FZD4	Ortiz-Masiá et al. (2020a)
SUCNR1	*In vitro In vivo* Human	HT29/TGFβ Hetero transplantation SUCNR1^−/−^CD B3: ↑Succinate, SUCNR1	↑	WNT	Ortiz-Masiá et al. (2020a)
AXL	*In vitro In vivo* Human	HT29 cells/TNFα TNBS IBD: ↑AXL	↑	ZEB/SNAIL	Boros et al. (2017)
TLR4	*In vitro In vivo*	HCT116 cells/LPS DSS TLR4^−/−^	↑	Cytokine expression	Jun et al. (2020)
AOPPs	*In vitro In vivo* Human	IEC6/AOPPs Rats/AOPPs, apocynin CD: ↑AOPPs	↑	PKC δ- NFκB	Xu X et al. (2017)
ZNF281	*In vitro In vivo* Human	HT29, IBD Biopsies/IFNγ,TNFα DSS IBD: ↑ZNF281	↑	SNAIL	Pierdomenico et al. (2018)
CHST15	*In vitro In vivo*	HCT116/TGFβ DSS CHST15 siRNA	↑	BMP7	Suzuki et al. (2016)
PTHLH	*In vivo* Human	TNBS overexpression PTH1R CD: ↑PTHLH and PTH1R	↑	PKA-Runx2	He et al. (2018)

#### 3.2.1 Cytokines and intestinal type-2 EMT

To begin with, TGFβ is the most important trigger of EMT (Yun et al., 2019). In the *canonical* pathway TGFβ induce the activation of the Small mothers against decapentaplegic (SMAD)2/3/4 complex which regulates the transcription of pro-EMT transcription factors (such SNAIL, ZEB or TWIST). *Non-canonical* (non-SMAD) TGFβ signalling mediates the induction of EMT through the activation of several kinases (ERK1/2, Akt, NFκB, TAK1, p38, JNK, ILK). Several studies indicate that aberrant signalling of TGFβ and its pathways lead to profibrotic EMT in IBD (Johnson et al., 2013; Di Gregorio et al., 2020). *In vitro* studies have shown that TGFβ induces the expression of IL13 via Dickkopf-homolog-1 (a WNT signalling antagonist) in IEC, and both cytokines exert a synergic effect on EMT activation (Frei et al., 2013; Scharl et al., 2013). In fact, the fourth European Crohn’s and Colitis Organisation (ECCO) guidelines state that the TGFβ/SMAD pathway activated by IL13 is a central process in the formation of intestinal fibrosis (Latella et al., 2014). Elevated secretion of IL13 is also associated with the expression of TNF-like cytokine 1A (TL1A) in *vivo* models (Giuffrida et al., 2019), a factor that is also capable of promoting EMT via TGFβ/SMAD (Wenxiu et al., 2021), which endorses the role of IL13 in fibrosis. Indeed, TL1A expression is upregulated in both UC and CD patients (Arimura et al., 2014).

Other cytokines involved in triggering EMT in intestinal fibrosis in *vivo* models include IL22 (Delbue et al., 2021) and IL17A (H.-J. Zhang et al., 2018). IL17A is a pro-inflammatory cytokine, mainly produced and secreted by Th17 cells, which contribute to the fibrotic process in multiple organs (Ramani and Biswas, 2019). On the other hand, the role of IL22 (a member of the IL-10 family) is more controversial and seems to depend on the cell type or type of inflammatory trigger (Keir et al., 2020). For instance, in spite of high levels of IL22 seen in IBD, epithelial barrier dysfunction persists (Pelczar et al., 2016) and IL22 trigger EMT via ERK in some preclinical models (Delbue et al., 2021). Interferon gamma (IFNγ) is another cytokine implicated in the activation of EMT in IBD, which acting on macrophages activate WNT signalling pathway (Macias-Ceja et al., 2022).

To sum up, the cytokines released during chronic inflammation can create an environment conducive to EMT, triggering fibrotic processes and therefore the progression of the disease. In IBD fibrotic context, the main pro-EMT cytokines described are Th1, Th2 or Th17 (TGFβ1, TL1A, IL17A, IL13, IL22 and IFNγ), where TGFβ1 is the best characterized pro-EMT agent, capable of triggering the induction of other pro-EMT cytokines. In addition, cytokines can exert their pro-EMT role both directly on epithelial cells but also indirectly through macrophages, further amplifying their pro-fibrotic effect. So, the intricate interplay between cytokines, EMT, and fibrosis highlights the complexity of cellular processes and the importance of maintaining proper balance for healthy tissue repair and function.

#### 3.2.2 Immune system and intestinal type-2 EMT

Macrophages play an important role in intestinal fibrosis since they are capable of generating a profibrotic environment, triggering EMT or fibroblast activation, and perpetuating the disease (Lis-López et al., 2021). In this line, Lipopolysaccharide (LPS)-treated macrophages induce EMT through Sonic Hedgehog (SHH) signalling (Ghorbaninejad et al., 2022), while IFNγ -treated macrophages (Macias-Ceja et al., 2022) trigger EMT via the WNT/FZD4 pathway. SHH and WNT signalling are evolutionary conserved signalling pathways which play a regulatory role in gut development and homeostasis and are both related with tumor progression and fibrosis (Castellone and Laukkanen, 2017). SHH protein is highly expressed in IEC and is involved in the regulation of epithelial cell turnover. In the inflamed tissues of IBD patients, SHH signalling components are overexpressed and *in vitro* assays have shown that inhibition of epithelial SHH signalling exerts a dual protective effect against inflammation and EMT (Ghorbaninejad et al., 2022). On the other hand, the WNT signalling pathway plays a vital role in homeostasis and repair, and has also been related to intestinal fibrosis (Lewis et al., 2022) and penetrating behaviour in CD (McGregor et al., 2023). In relation to the modulation of intestinal EMT, it has been described that IEC cocultured with IFNγ -treated macrophages (Macias-Ceja et al., 2022) or WNT2b (Ortiz-Masiá et al., 2020a) trigger EMT via the FZD4 receptor. These *in vitro* assays have been endorsed by the fact that IFNγ, the IFNγ receptor and the WNT2b/FZD4 pathway are overexpressed in CD patients with stenotic and/or penetrating behaviour (Ortiz-Masiá et al., 2020a; Macias-Ceja et al., 2022).

Other proteins involved in immune responses in fibrotic conditions are Toll-like receptors (TLRs) and TAM receptors. Within the family of TAM receptors (pleiotropic negative regulators of the immune system), the AXL receptor has been specifically implicated in the regulation of cell motility and EMT in IBD. In both *in vivo* models and in IBD tissue, inflammation has been shown to trigger AXL overexpression in epithelial cells and macrophages which is accompanied by an increase in the EMT markers (VIMENTIN, ZEB2 and SNAIL) (Boros et al., 2017; Boros et al., 2018). However, the TLR4 receptor (a facilitator of inflammatory responses through maturation of innate immunity) also triggers intestinal EMT in *vivo* and *in vitro* models (Jun et al., 2020). The actions of both receptors are mediated through profibrotic NFκB signalling, which may partly explain that both trigger EMT (Lemke and Rothlin, 2008; Jeong and Lee, 2011).

In summary, macrophages and immune receptors (such TLRs and TAM receptors) in IBD exert their pro-fibrotic role through the activation of pro-EMT pathways related with development and homeostasis regeneration (WNT or SHH pathways) and NFκB signaling, respectively. These results support the close relationship between inflammation and fibrosis in IBD, such that imbalance between immune responses and tissue repair processes potentially promotes fibrosis.

#### 3.2.3 Oxidative stress and intestinal type-2 EMT

Oxidative stress is a hallmark of IBD and there is a well-established link between ROS production, oxidative stress, and the activation of pro-fibrotic growth factors and cytokines, suggesting the existence of feedback as well as feed-forward cycle in intestinal fibrosis (Latella, 2018). In relation to EMT-type 2, oxidative stress is considered a stimulus in lung fibrosis (Cheresh et al., 2013). In IBD, the accumulation of advanced oxidation protein products (AOPPs) promote inflammation and fibrosis formation by activating cellular oxidative stress (Balmus et al., 2016). AOPPs correlate with the expression the EMT markers in intestinal fibrosis, and *in vitro* and *in vivo* administration of AOPPs induces EMT via the protein kinase C δ isoform (PKC δ) that triggers NFκB pathway (Xu X et al., 2017).

Another molecule implicated in oxidative stress is succinate. Succinate levels and its receptor SUCNR1 are increased in CD patients and correlate with EMT markers. (Macias-Ceja et al., 2019; Ortiz-Masiá et al., 2020a) (Table 2). Succinate is an important metabolite at the cross-road of several metabolic pathways, also involved in the formation and elimination of reactive oxygen species (ROS), and succinate accumulation contributes to oxidative stress and mitochondrial ROS production (Zhang et al., 2021). SUCNR1 is activated by succinate when this metabolite is secreted to the extracellular milieu after accumulation inside cells suffering metabolic alterations provoked by inflammatory mediators. In IBD models, succinate and SUCNR1 are capable of triggering EMT through the WNT pathway *in vitro* and *in vivo* (in a heterotopic intestinal transplant model of fibrosis in SUCNR1−/− mice) (Ortiz-Masiá et al., 2020a) (Table 2).

#### 3.2.4 Other molecules related with intestinal type-2 EMT

Other novel molecules that have been linked to intestinal EMT are the transcription factor ZNF281 (Pierdomenico et al., 2018), the enzyme carbohydrate sulfotransferase 15 (CHST15) (Suzuki et al., 2016) and the parathyroid hormone–like hormone (PTHLH) (He et al., 2018). The novel factor ZNF281is overexpressed in IBD patients and required for the induction of SNAIL-dependent EMT. CHST15 is an enzyme biosynthesizing chondroitin sulphate E which binds to various proinflammatory and profibrotic mediators and is known to create local fibrotic lesions. In fact, STNM01, a synthetic double-stranded RNA oligonucleotide directed against CHST15, is currently in a Phase 1 Clinical Study (safety) in CD patients (Suzuki et al., 2017). Finally, PTHLH is a multifunctional peptide implicated in fibrosis formation (Ardura et al., 2010), and induces EMT in IEC of CD patients by modulating protein kinase A (He et al., 2018).

#### 3.2.5 Molecular mechanisms implicated in the downregulation of type-2 EMT in IBD

At the other end of the spectrum of the molecular mechanisms involved, there are the molecules or pathways that favour mesenchymal epithelial transition (MET) or prevent EMT (Table 3) (Figure 2). For instance, bone morphogenic protein 7 (BMP7) is a member of the TGFβ family and prevents TGFβ-induced EMT *in vivo* and *in vitro* due to its ability to counteract the profibrotic effect of TGFβ (Flier et al., 2010). Other molecules that appear to downregulate EMT are the peroxisome proliferator-activated receptor (PPAR)γ and SIRT1 (a class III lysine deacetylase) as their ablation has been shown to exacerbate EMT in *vivo* models of intestinal fibrosis (Di Gregorio et al., 2017; Chen et al., 2021). PPARγ is a well-known inhibitor of TGFβ-induced EMT by antagonizing SMAD3 function (Reka et al., 2010), and PPARγ activators seem to reverse intestinal fibrosis (Di Gregorio et al., 2017; Xu S et al., 2017). SIRT1 is an enzyme that plays a crucial role in aging and chronic diseases. It functions by deacetylating several transcription factors, thereby regulating various pathways. One such pathway in which SIRT1 has been implicated is intestinal fibrosis-associated EMT (Chen et al., 2021), in which deacetyl SMAD4 and subsequently block the signalling TGFβ (Simic et al., 2013). Finally, it has been shown that the vitamin D receptor (VDR) inhibits EMT modulating the mitochondrial respiratory chain. VDR deficiency causes mitochondrial dysfunction in the intestinal epithelium and promotes fibrosis by upregulating the EMT pathway. In fact, low levels of VDR have been detected in patients with CD (Yu et al., 2021).

**TABLE 3:** Molecular mechanisms implicated in the downregulation of type-2 EMT in IBD samples, and *in vivo* and *in vitro* IBD related models. The symbol “/” indicates treatment. Bone morphogenic protein-7 (BMP7); Dextran sodium sulfate (DSS); Glycogen synthase kinase (GSK); Intestinal epithelial cell (IEC); Peroxisome proliferator-activated receptor (PPARγ); Silent information regulator 1 (SIRT1); Transforming growth factor (TGF); Trinitrobenzene sulfonic acid (TNBS); Vitamin D (VD); Vitamin D receptor (VDR).

Protein	Type of study		EMT	Molecular mechanism in EMT	References
BMP7	*In vivo*	TNBS VillinCre; R26Rosa-lox-STOP-lox-LacZ mice (trace IECs)	↓	BMP7 is an inhibitor of TGFβ	Flier et al. (2010)
GSK3β/PPARγ	*In vivo*	DSS/GW9662 (PPARγ inhibitor) DSS/GED-0507-34 Levo (PPARγ agonist)	↓	GSK3β activate PPARγ signaling	Di Gregorio et al. (2017)
SIRT1	*In vitro In vivo*	IEC6/TGFβ TNBS SIRT1^−/−^	↓	Blocks TGFβ through SMAD4 and KDM4-DBC1axis	Simic et al. (2013), Chen et al. (2021)
VDR	*In vitro In vivo* Human	HT29, CCDA18Co cells/VD TNBS VDR^−/−^CD: ↓VDR	↓	Epithelial mitochondria-mediated EMT	Yu et al. (2021)

Dysregulated autophagy is a hallmark of IBD (Shao et al., 2021), and while its role in intestinal fibrosis is controversial (Macias-Ceja et al., 2023), several studies support that autophagy stimulation may be an antifibrotic strategy (Cosin-Roger et al., 2019; Zeng et al., 2022). It has been reported that autophagy activation can suppress EMT by crosstalking with various signaling pathways (e.g., WNTs, NF-kB, TGF-β, NOTCH and Fibrinogen-like protein 1 (FGL-1) signaling pathways) (H.-T. Chen et al., 2019; Gao et al., 2023; Hill et al., 2019). Indeed, in lung fibrosis, autophagy inhibition-induced EMT of alveolar epithelial cells contributes to fibrosis not only by affecting the epithelial phenotype but also via aberrant epithelial–fibroblast crosstalk (Hill et al., 2019). In intestinal fibrosis, Zeng’s work showed that autophagy stimulation inhibited EMT in a DSS model, ameliorating intestinal fibrosis (Zeng et al., 2022) (Table 6).

EMT and its converse, MET, are integral stages of many physiologic processes (e.g., wound healing) and as such, are tightly coordinated. In wound healing, EMT as a response to injury can be beneficial. However, if the wound healing process is exaggerated, it may lead to fibrosis. Carrying this idea over to IBD, intestinal epithelial cells are chronically immersed in a pro-EMT factor-rich environment that disrupts the EMT/MET imbalance. In the previous sections, numerous pro-EMT factors have been described that are increased in tissues from patients with IBD, such as cytokines (IL13, TGFβ, TL1A, or IFNγ), pathways involved in development and homeostasis regeneration (WNT or SHH pathways), among others (Table 2). But in addition, there are processes such as the inhibition of autophagy that would also contribute to the imbalance, favoring and further perpetuating intestinal fibrosis in IBD.

### 3.3 Epigenetic factors in intestinal EMT

Epigenetic modifications, which include DNA methylation, or ncRNA molecules, can play a crucial role in regulating EMT. ncRNAs have been proved to participate in the fibrotic diseases of multiple organs (e.g., liver diseases, myocardial fibrosis, and renal fibrosis). The ncRNAs involved in fibrotic diseases mainly consist of microRNAs (miRNAs), long noncoding RNAs, and circular RNAs (circRNAs). NcRNAs modulate the function of mesenchymal cells, inflammatory cascades, ECM, and microbiota via mechanisms of endogenous RNA competition, RNA transcription regulation, protein sponges, and translation regulation (Zhou et al., 2021). The role of microRNAs in the intestinal EMT has been extensively studied (Boros et al., 2017; Boros and Nagy, 2019). Most of the works analyse the role of miRNAs in type-3 EMT, that is, in the progression of colorectal cancer in IBD. However, miR-200b has been shown to be effective in preventing EMT and in alleviating intestinal fibrosis. miR-200b functions by targeting the 3′untranslated region (UTR) of ZEB1 and ZEB2 mRNAs, leading to translational repression (Chen et al., 2012; 2013; Zidar et al., 2016) (Table 4). Indeed, a downregulation of the miR-200 family has been described in patients with IBD (Zidar et al., 2016). Other microRNAs with a potential role in the type-2 EMT associated to IBD are miR-199a, miR-34a, miR-155-5p, miR-146a-3p, and miR-213p (Table 4). In the inflamed tissue of patients with IBD, it has been described that miR-199a and miR-34a expression is reduced and is accompanied by a high expression of AXL tyrosine kinase receptor (Boros et al., 2018). It is interesting that, in a similar way, both miRNA downregulate AXL in lung, colorectal, and breast cancer models (Mudduluru et al., 2011). More recently, miR-155-5p, miR-146a-3p and miR-213p expression have been shown to be inversely correlated with E-cadherin gene expression in tissue biopsies from CD patients (Guz et al., 2020), but further investigations are necessary to establish their specific mechanisms. Finally, a recent study has shown that Circ_0001666, a circRNA, controls EMT by regulating the stability of BMP7 mRNA through its interaction with Serine/arginine-rich splicing factor 1 (SRSF1), thus promoting fibrosis in pediatric CD. Indeed, the expression of circ_0001666 is upregulated in CD pediatric tissues (Table 4) (Li et al., 2023).

**TABLE 4:** Genes involved in intestinal EMT and their non-coding RNA (ncRNA) regulators in IBD. The symbol “/” indicates treatment. Bone morphogenic protein-7 (BMP7), E-cadherin (CDH1), N-cadherin (CDH2), Crohn’s disease (CD); Dextran sodium sulfate (DSS); Epithelial Growth factor (EGF); Fibroblast growth factor (FGF); Intestinal epithelial cell (IEC); Serine/arginine-rich splicing factor 1 (SRSF1); Transforming growth factor (TGF); Trinitrobenzene sulfonic acid (TNBS); Zinc-finger E-box-binding (ZEB).

ncRNA	Target	Type of study		Effect in EMT		References
miR-200	ZEB1/SMAD2	*In vitro* Human	IEC6/TGFβ1 IBD: ↓ miR-200b	↓	↑CDH1 ↓Vimentin	Chen et al. (2012), Chen et al. (2013), Zidar et al. (2016)
miR-199a and miR-34a	AXL	Human	IBD: ↓ miR-199a and miR-34a	↓	↓ZEB2, SNAIL1	Mudduluru et al. (2011), Cho et al. (2016), Boros et al. (2017), Boros et al. (2018)
miR-155-5p, miR-146a-3p, miR-213p	CDH1	Human	CD: ↑miR-146a3p, miR-155-5p and miR-213p	↑	↓CDH1	Guz et al. (2020)
Circ_0001666	SRSF1/BMP7	*In vitro* Human	IEC/TGFβ1 CD: ↑ Circ_0001666	↑	↓CDH1 ↑Vimentin, SNAIL, CDH2	Li et al. (2023)

In relation to DNA methylation, several studies suggest a link between EMT and UC progression/prognosis (type-3 EMT), specifically in the context of epigenetic modifications of EMT-related genes (Saito et al., 2011; Wang et al., 2012; Tahara et al., 2014; Zhao et al., 2015). The findings suggest that hypermethylation of CDH1, CDH13, NEUROG1, CDX1, and miR-1247 are associated with inflammatory rectal samples compared to non-inflammatory mucosa in control samples. Furthermore, this hypermethylation is correlated with a more severe clinical phenotype in UC patients.

### 3.4 Microbiome as inductor of intestinal EMT

Several studies indicate that gut microbiota plays crucial roles in fibrosis. In several animal models, microbes initiate or perpetuate gut fibrosis (Rieder, 2013). In CD fibroblasts, there is an increased expression of several TLRs that can be activated by perceiving microbial components and promote transdifferentiation (Zorzi et al., 2015). However, there is little direct evidence so far on the possible involvement of EMT in microbiome-induced intestinal fibrosis, and the studies are indirect and involve TGFβ changes. In this line, antibiotic treatment significantly inhibits TGFβ1 or the injection of faecal material or extracts from anaerobic bacteria into the bowel wall induced fibrosis and increased levels of TGFβ1 (Mourelle et al., 1998).

Some enteric pathogens have been shown to be able to modulate EMT in IECs (Table 5), such us *Helicobacter pylori* (Yin et al., 2010; Ouyang et al., 2021), *Citrobacter rodentium* (Chandrakesan et al., 2014), *Escherichia coli* (Cane et al., 2010) or *Clostridium butyricum* (Zhang et al., 2023), however these infections are not related with intestinal fibrosis.

**TABLE 5:** Microbiome as inductor of intestinal EMT in IBD. Hypoxia inducible factor (HIF); vascular endothelial growth factor (VEGF); m^6^A methyltransferase (METTL3).

Microbiome	Effect in EMT		References
*Helicobacter pylori*	↑	AKT/GSK3β signaling	Yin et al. (2010), Ouyang et al. (2021)
*Citrobacter rodentium*	↑	WNT/NOTCH signaling	Chandrakesan et al. (2014)
*Escherichia coli*	↑	HIF1α/IL8/VEGF/TWIST1	Cane et al. (2010)
*Clostridium butyricum*	↓	METTL3	Zhang et al. (2023)

### 3.5 EMT as a therapeutic target in intestinal fibrosis associated with IBD

Although organ fibrosis was considered an irreversible process, it is now known to be a dynamic process with the potential for reversibility and restoration of near-normal tissue architecture and organ function. Several approaches (antioxidants, inhibition of fibrotic signalling pathways, stem cell therapies, modulation of fibrogenic cells or anti-inflammatory targets) have shown anti-fibrotic effects in animal models of organ fibrosis (Horowitz and Thannickal, 2019; Lurje et al., 2023), and some of them are currently approved for human use in certain fibrotic diseases (Bocchino et al., 2023). Given the potential role of EMT in IBD-associated fibrosis, several strategies targeting EMT have been explored as potential therapeutic approaches for IBD. In this section, we review the main products tested in *vivo* and *in vitro* models of intestinal fibrosis, whose main mechanism is to modulate intestinal EMT (Table 6).

**TABLE 6:** EMT as a therapeutic target in intestinal fibrosis associated to IBD. Activating Protein-1 (AP1); Crohn’s disease (CD); Dextran sodium sulfate (DSS); Hypoxia inducible factor (HIF); Intestinal epithelial cell (IEC); Ionizing radiation (IR); Nuclear Factor kB (NFκB); Peroxisome proliferator-activated receptor (PPARγ); Recombinant human bone morphogenic protein-7 (rhBMP7); Signal transducer and activator of transcription (STAT); Transforming growth factor (TGF); Trinitrobenzene sulfonic acid (TNBS); Zinc-finger E-box-binding (Zeb).

Product	Target	Type of study		References
Exopolysaccharide *Bacillus subtilis*	NFΚb, STAT3 Immune cell infiltration	*In vivo*	DSS	Chung et al. (2021)
Curcumin	PPARγ activator	*In vivo*	TNBS	Xu S et al. (2017)
Silibinin	TGFβ1	*In vivo/vitro*	IR, CD	Kim et al. (2017)
Abelmoschus manihot	TGFβ1	*In vitro*	IEC6	Yang et al. (2018)
Halofuginone	TGFβ/Smad	*In vitro*	IPEC-J2 cells	Duan et al. (2020)
Atractylenolide III	TGFβ1	*In vitro*	IEC6	Huang et al. (2022)
Wu-Mei-Wan	NFκB, STAT3 TGFβ/Smad Wnt/β-catenin	*In vivo*	TNBS	Wu et al. (2020)
Forsythia koreana	AP1, NFκB, and STAT1/3 macrophages	*In vivo/vitro*	DSS, RAW264.7 cells	Kim et al. (2019)
HLJ2 (berberine)	NFκB	*In vivo*	DSS	Song et al. (2020)
Artemisinin	ERK/MYD88 signaling M2 macrophages	*In vivo/vitro*	DSS, CD, RAW264.7 cells	Huai et al. (2021)
Xue-Jie-San	Autophagy stimulation	*In vivo*	TNBS	Gao et al. (2023)
Resolvin D1	Autophagy stimulation	*In vivo*	DSS	Zeng et al. (2022)
Mesenchymal cells	TGFβ/SMAD	*In vivo*	TNBS	Lian et al. (2018)
miR-200b	Zeb1/2	*In vivo/vitro*	TNBS, IEC6	Yang et al. (2017b)
A83-01	TGFβ1	*In vitro*	Caco2	Ghorbaninejad et al. (2023)
rhBMP7	TGFβ1	*In vivo*	TNBS	Flier et al. (2010)
GED-0507-34 Levo	PPARγ activator	*In vivo*	DSS	Di Gregorio et al. (2017), Pompili et al. (2023)
*Saccharomyces boulardii*	HIF1/2	*In vivo*	DSS	Zhou et al. (2018)
AMA0825	Rho Kinases	*In vivo*	DSS	Holvoet et al. (2017)
Xue-Jie-San	Autophagy stimulation	*In vivo*	TNBS	Gao et al. (2023)

The use of natural products and derivatives, including those derived from traditional Chinese medicine, has gained attention in the field of fibrosis research. Many natural products have been investigated, in *vivo* models of IBD, for their potential anti-fibrotic effects by targeting EMT pathways. Most products have TGF-mediated EMT as their primary target: both *canonical* (curcumin (Xu S et al., 2017), silibin (Kim et al., 2017), *Abelmoschus manihot* (Yang et al., 2018), Wu-Mei-Wan (Wu et al., 2020), halofuginone (Duan et al., 2020), and Atractylenolide III (Huang et al., 2022)) and *non-canonica*l downstream pathways (Wu-Mei-Wan (Wu et al., 2020), *Forsythia koreana* (T.-W. Kim et al., 2019), HLJ2 (Song et al., 2020) and Artemisinin (Huai et al., 2021)).

Several studies support that autophagy stimulation may be an antifibrotic strategy (Cosin-Roger et al., 2019; Zeng et al., 2022). In this sense, Xue-Jie-San, a traditional Chinese herb, protects against EMT-mediated fibrosis through the stimulation of autophagy, blocking the NOTCH1 and FGL1 signalling pathways (Gao et al., 2023). In fact, NOTCH signalling is a profibrotic pathway that has been little studied in IBD-related intestinal fibrosis (Marti-Chafer et al., 2023). Other molecule that prevents intestinal EMT by stimulating epithelial autophagy is resolvin D1, an omega-3 polyunsaturated fatty acid (Zeng et al., 2022).

There is growing evidence suggesting that the interactions between the gut microbiota and the host can influence EMT and contribute to the development of intestinal fibrosis (Table 5). Modifying the microbiota through dietary interventions has emerged as a potential strategy to influence EMT and attenuate fibrosis in various intestinal fibrotic models (Yang et al., 2017a; Zhou et al., 2018; Chung et al., 2021). Similarly, cell therapy as a control mechanism for EMT has also been analysed in intestinal fibrosis. Indeed, TNBS models have shown that: mesenchymal stem cell (MSC) exert anti-fibrogenic activity by regulating the inflammatory environment, inhibiting the TGFβ/SMAD signalling pathway and ameliorating EMT (Lian et al., 2018). Likewise, the delivery of miR-200b through bone marrow MSC-derived microvesicles inhibits EMT and ameliorate fibrosis (Yang et al., 2017b).

Finally, there are other synthetic molecules tested in preclinical models that inhibit intestinal EMT which have a promising future: the recombinant human BMP7 (rhBMP7), GED-0507-34 Levo, AMA0825 or A83-01. In preclinical studies, rhBMP7 has demonstrated the ability to inhibit EMT and attenuate fibrosis in various organs (Weiskirchen and Meurer, 2013), including the intestine (Flier et al., 2010). It exerts its anti-fibrotic effects by antagonizing TGFβ-induced EMT and promoting tissue repair and regeneration. GED-0507-34 Levo is an orally active synthetic compound and a selective agonist of PPARγ that has been shown to inhibit EMT, reduce inflammation, and ameliorate fibrosis in a DSS model (Di Gregorio et al., 2017; Pompili et al., 2023). In fact, GED-0507-34 is in a Phase 2 clinical trial in subjects with active, mild-to-moderate UC (ClinicalTrials.gov Identifier: NCT02808390). AMA0825, a Rho kinase inhibitor, is a synthetic small molecule that has been studied in intestinal fibrosis due to its potential effects on EMT and autophagy (Holvoet et al., 2017). Finally, A83-01, a new type I receptor ALK5 kinase inhibitor molecule, that in an *in vitro* assay blocks TGFβ-induced EMT (Gao et al., 2023).

In summary, there have been multiple trials focused on targeting EMT to manage intestinal fibrosis in the context of IBD. While many of these trials are still in the preclinical stages, some have progressed to clinical trials, such as the trial involving GED-0507-34.

## 4 Other roles of epithelial cells in intestinal fibrosis: lessons from other fibrotic tissues

Epithelial cell injury and death are common events in inflammatory diseases, such us UC and CD, but they have been only recently recognized as drivers of fibrosis. For instance, an increasing number of studies have linked necroptosis (a form of programmed necrosis) to inflammation and fibrosis in renal, liver, heart or lung fibrosis (Liu et al., 2022). Cell products released by cells undergoing necrosis (passive, programmed, or after apoptosis) are called damage-associated molecular patterns (DAMPs), that can directly activate profibrotic responses of immune cells or nonimmune cells (epithelial cells, endothelial cells, and fibroblasts) triggering fibrosis (Liu et al., 2022). In addition, epithelial cells contain a myriad of intracellular substances normally not recognized by the immune system but, during cell necrosis, they are passively released in the surrounding microenvironment and trigger inflammation. These responses may represent a novel fibrotic pathogenic component of IBD since epithelial damage is a typical feature of both UC and CD.

DAMPs are classified into molecules that perform noninflammatory functions or alarmins. The noninflammatory DAMPS in living cells (such as high-mobility group box 1, HMGB1) can acquire immunomodulatory properties when released, secreted, modified, or exposed on the cell surface during cellular stress, damage, or injury. On the other hand, alarmins alert the immune system and trigger a sterile inflammatory response (such as IL1α, S100A8, and IL33) (Kaczmarek et al., 2013). IBD tissue releases calprotectin (S100A12, S100A8/S100A9 complexes) and HMGB1 which serve as faecal biomarkers of intestinal inflammation (Nanini et al., 2018). The role of several necroptotic DAMPs and their receptors have been described in the main fibrotic diseases, except in intestinal fibrosis associated to IBD (Liu et al., 2022), where further studies are needed. In this line, Scarpa and collaborators have reported that epithelial cell-derived DAMPS (IL1α) elicit a potent proinflammatory cytokine response from human intestinal fibroblasts. Fibroblasts would act as first responders to products of IECs necrosis due to their anatomical proximity (Scarpa et al., 2015). Necroptotic DAMP receptors have also been reported to promote fibrosis. In IBD, genetic knockout TLR4 (a well-known necroptotic receptor) can alleviate systemic inflammation and tissue fibrosis in intestine, via cytokine expression and EMT (Jun et al., 2020). In the same line of the role of epithelial cells as sources of profibrotic ligands, accumulated data regarding pulmonary fibrosis show that EMT transdifferentiation does not occur completely Rather, the EMT cells act as sources of soluble ligands that favour the transdifferentiation of fibroblasts (Hill et al., 2019; Yue et al., 2022). This phenomenon has also been observed in IBD by Zeng and collaborations, who found that the co-culture of EMT cells with intestinal fibroblast induced fibroblast activation (Zeng et al., 2022).

On the other hand, there is the epithelial-immune crosstalk described in both pulmonary and cutaneous fibrosis (Planté-Bordeneuve et al., 2021; Rosenblum and Naik, 2022). Interactions between epithelium and the immune system involve a tight regulation to prevent inappropriate reactions. Recent data regarding pulmonary fibrosis suggest a two-way process, so that epithelial cells’ biology and their crosstalk with immune cells and microbes may trigger aberrant pro-fibrotic signalling (Planté-Bordeneuve et al., 2021). Intestinal epithelium and immunity have been implicated in the pathogenesis and disease course of IBD. However, consequences of their abnormal interplay in fibrosis remain unknown.

## 5 Conclusion and remarks

Intestinal fibrosis associated with IBD is a complex condition that has been the focus of ongoing research in the last decade, especially in CD. The role of epithelial cells in the pathogenesis of intestinal fibrosis has been widely studied and currently, one of the main cellular mechanisms involved in intestinal fibrosis is the epithelial-mesenchymal transition. Given the potential role of EMT in IBD-associated fibrosis and the lack of pharmacological therapies for this condition, several novel strategies targeting EMT have been explored. It is important to note that these therapies hold promise, but more research is needed to determine their efficacy, safety, and long-term outcomes in the IBD setting.

In relation to future perspectives, more in-depth studies are required on the role of the microbiota and epigenetics in EMT-mediated intestinal fibrosis since the available works are focused either on the oncological progression of the disease or not directly related with intestinal fibrosis. Similarly, outside the epithelial transition, evidence for the involvement of epithelial-immune or epithelial-mesenchymal crosstalk in IBD fibrosis is limited. Determining the exact contribution of these mechanisms is challenging, as they are at the crossroads of multiple regulatory networks. Nonetheless, in-depth understanding of the epithelial contribution to the fibrotic paradigm will help to design more specific and effective anti-fibrotic therapies.
